# Assessment of the primary rotational stability of uncemented hip stems using an analytical model: Comparison with finite element analyses

**DOI:** 10.1186/1749-799X-3-44

**Published:** 2008-09-25

**Authors:** Maria E Zeman, Nicolas Sauwen, Luc Labey, Michiel Mulier, Georges Van der Perre, Siegfried VN Jaecques

**Affiliations:** 1 Katholieke Universiteit Leuven (K.U.Leuven), Division of Biomechanics and Engineering Design (BMGO), Celestijnenlaan 300C, 3001 Heverlee, Belgium; 2 University Hospitals Leuven (UZ Leuven), Orthopaedics Section, Weligerveld 1 blok 2 – bus 7001, 3212 Pellenberg, Belgium; 3 Katholieke Universiteit Leuven (K.U.Leuven), Department of Dentistry, Oral Pathology and Maxillo-Facial Surgery, BIOMAT Research Cluster, Kapucijnenvoer 7 – bus 7001, 3000 Leuven, Belgium

## Abstract

**Background:**

Sufficient primary stability is a prerequisite for the clinical success of cementless implants. Therefore, it is important to have an estimation of the primary stability that can be achieved with new stem designs in a pre-clinical trial. Fast assessment of the primary stability is also useful in the preoperative planning of total hip replacements, and to an even larger extent in intraoperatively custom-made prosthesis systems, which result in a wide variety of stem geometries.

**Methods:**

An analytical model is proposed to numerically predict the relative primary stability of cementless hip stems. This analytical approach is based upon the principle of virtual work and a straightforward mechanical model. For five custom-made implant designs, the resistance against axial rotation was assessed through the analytical model as well as through finite element modelling (FEM).

**Results:**

The analytical approach can be considered as a first attempt to theoretically evaluate the primary stability of hip stems without using FEM, which makes it fast and inexpensive compared to other methods. A reasonable agreement was found in the stability ranking of the stems obtained with both methods. However, due to the simplifying assumptions underlying the analytical model it predicts very rigid stability behaviour: estimated stem rotation was two to three orders of magnitude smaller, compared with the FEM results.

**Conclusion:**

Based on the results of this study, the analytical model might be useful as a comparative tool for the assessment of the primary stability of cementless hip stems.

## Background

The term primary stability refers to the inducible displacement between an implant and the surrounding bone, under physiological loading of the implant in the early postoperative stage, when osseointagration has not yet occurred. Sufficient primary stability is a prerequisite for the long term success of cementless total hip replacements (THRs). Various authors suggest that osseointegration becomes unlikely at micromotions larger than 150 *μ*m [1,2]. Instead, a fibrous interface tissue will be formed, which does not give adequate support to the implant. This will compromise the endurance of the implant fixation and may lead to aseptic loosening, which is the primary cause of failure in cementless THR [1,3].

The primary stability of cementless hip implants has been investigated extensively, in vitro as well as numerically. Finite element (FE) studies have contributed to the research on primary stability in several ways. Some studies have investigated the influence of certain factors on the primary stability, e.g. bone quality [4], loading conditions [5], amount of press-fit [6] and the presence of gaps at the bone-implant interface [7]. Other FE studies have evaluated the primary stability of new prosthetic designs [8,9]. It has also been suggested to use finite element modelling (FEM) in preoperative planning of THRs [10] to quantify the expected primary stability. However, the introduction of FE methods into the preoperative environment would require specialised software and high performance computing hardware to keep the runtime of the simulation within acceptable limits. This would considerably raise the cost of the procedure. Fast assessment of the primary stability is even more important when custom-made stems are designed intraoperatively, based on the geometry of the reamed cavity. Furthermore, a protocol would be needed to automatically generate accurate patient-specific models, to account for the inter-subject variability [11]. The development of such a protocol is far from evident, and it will also result in higher costs and longer runtimes.

In vitro studies usually consider the micromotion at the interface between the prosthesis and the bone under physiological loading conditions [12-17]. However, methods of measurement, points of measurement, loading conditions, and the designs tested have varied among different studies. This has limited the comparability of these studies. A wide range of inducible displacements was found for comparable loading conditions: for instance, when loading conditions simulating stair climbing were applied, micromotions were found in the range of 10–50 *μ*m [17], 10–280 *μ*m [12], 10–380 *μ*m [13] and 240–1540 *μ*m [16]. Considering this large experimental variability in measuring micromotion, several authors have suggested that a theoretical approach could help in determining the potential stability of different stem designs. It would be very useful to be able to make statements about the primary stability of a hip stem without the need for measurements.

Torsional loading (e.g. stair climbing) has been shown to cause the largest displacements at the bone-implant interface [13,18]. Therefore, large torsional loads, e.g. stair climbing, must be avoided in the first postoperative months. New implant designs should aim for a good resistance against axial rotation, to ensure sufficient primary stability.

Several in vitro studies have investigated the influence of the stem geometry on the primary stability, by comparing the magnitudes of motion between different stem types [13,17,19,20]. These studies pointed out that the geometry of the stem significantly affects the primary stability and can be important in the prevention of excessive micromotion. Very few attempts have been made so far to define a parameter able to quantify the potential stability that can be achieved with a specific stem design. Ruben et al. proposed an optimisation strategy to design new hip stems, based on two objective stability functions [21]. The first one is a function of tangential displacement at the bone-stem interface, the second one is a function of normal contact stresses. A mapping of the relative displacements and the normal contact stresses at the bone-stem interface is obtained using FEM. To the authors' knowledge, currently no stability characteristics have been proposed without the need for FEM.

A parameter characterising the potential primary stability of a hip stem could also be of great value in pre-operative planning of THRs. It could provide the surgeon with objective information to help him choose the best stem type in patient-specific cases. The traditional way of planning a THR is to superpose transparent templates of prostheses onto a radiograph of the hip joint, to determine the most suitable stem size and type [22]. However, this procedure does not provide the surgeon with much information about the quality of the surrounding bone and a radiograph provides only limited geometrical information. A study by Viceconti et al. has shown that, by using a preoperative planning system, the implanted stem geometry more often corresponds to the planned stem geometry than when templates are used [23]. Furthermore, the difference in planning result is smaller among different surgeons. Currently, there is no consensus about the best criterion to predict the long term success of a THR. Therefore, the current preoperative planning systems rely on very divergent criteria as a measure of the expected success [24-26]: HipOp, a planning system developed by Viceconti et al., provides the user with two analysis modules to assess the bone quality around the implant [24]; a planning system developed by Duda et al. [25] on the other hand estimates the joint contact force, based on a musculoskeletal model; and Benedetti et al. presented two computer-based tools to be used in preoperative planning of THRs [26]. Both tools are based on gait analysis: one tool aims at restoring correct joint motion, while the other one considers the lever arms of the abductor muscles and leg-length discrepancy. Although it has been shown that good primary stability is essential to achieve long term functionality of cementless implants [1,2,27], no quantitative relationship has yet been established between the primary stability and long term results. However, a parameter quantifying the primary stability could be a good predictor for the long term results, and might thus be useful as a criterion for the expected success of THRs in a preoperative planning system [23].

At the department of orthopaedic surgery of the Leuven university hospitals, an intraoperatively custom-made prosthesis (IMP) system is used, based on the theory that a THR stem with optimal fit and fill of the intramedullary canal will resist the daily loads on the hip better than standard stems [28]. However, a large variety of stem geometries is obtained with this technique, and sufficient primary stability of the stems is not guaranteed.

This study proposes an algebraic formula, which allows a fast estimation of the primary stability of a given implant design under torsional loading. The analytical formula is based on a straightforward mechanical model and the principle of virtual work. The suitability of the analytical model as a measure for the primary stability was investigated and confirmed for five custom-made stem designs using FEM.

## Methods

### Analytical model

The resistance against axial rotation was evaluated through the proposed analytical model which is explained as follows. The hip stem is considered as a rigid body. The femoral cavity is assumed to be perfectly filled and fitted by the prosthesis stem. The geometry of the stem surface is described by a point cloud and can be divided in a set of triangles (a so-called STL-description). The stem is supposed to be in contact with supporting bone with equal thickness over the entire surface, and the outer bone surface is assumed to be rigidly fixed. The bone behaves as a linear elastic material. We assume that the resistive forces $\vec{F_{i}}$ from the supporting bone act at the centre of gravity C_i _of each triangle and are perpendicular to its surface. Furthermore, frictional forces are neglected (although friction could be implemented in a later stage):

(1) $$\vec{F_{i}}=-F_{i}\cdot \vec{n_{i}}\text{ for each triangle i}$$

The normal vector is directed to the outside of the prosthesis surface, while the force is of course directed towards the surface, hence the minus sign.

**Figure 1:**
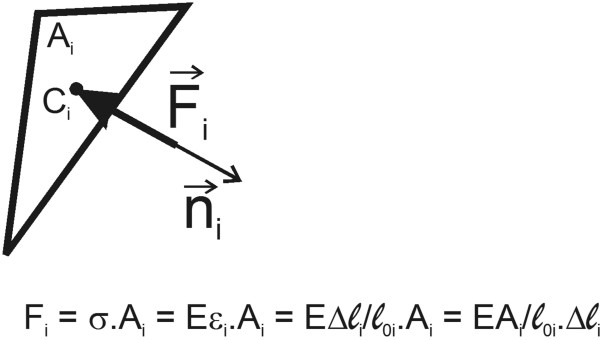
One element of the prosthesis surface supported by bone.

We assume that the resistive force is proportional to the normal displacement at the bone-implant interface. F_i_, the magnitude of the resistive force, can be written as (figure 1):

(2) $$F_{i}=\left(\frac{EA_{i}}{\ell_{0i}}\right)\cdot \Delta \ell_{i}$$

Where E is the Young's modulus of bone, A_i _is the area of triangle i, ℓ_0i _is the thickness of the supporting bone when it is undeformed and Δℓ_i _is the change in thickness of the bone due to displacement of the prosthesis. Δℓ_i _can also be written as:

(3) $$\begin{array}{l} \Delta \ell_{i}=\vec{\Delta r_{Ci}}\cdot \vec{n_{i}}\text{ when }\vec{\Delta r_{Ci}}\cdot \vec{n_{i}}>0\text{ and}\\ \Delta \ell_{i}=0\text{ when }\vec{\Delta r_{Ci}}\cdot \vec{n_{i}}\leq 0 \end{array}$$

with $\vec{\Delta r_{Ci}}$ the displacement of the centre of gravity of triangle i. $\vec{\Delta r_{Ci}}$ and $\vec{n_{i}}$ should have the same sense, because otherwise the prosthesis becomes loose in point C_i_.

**Figure 2:**
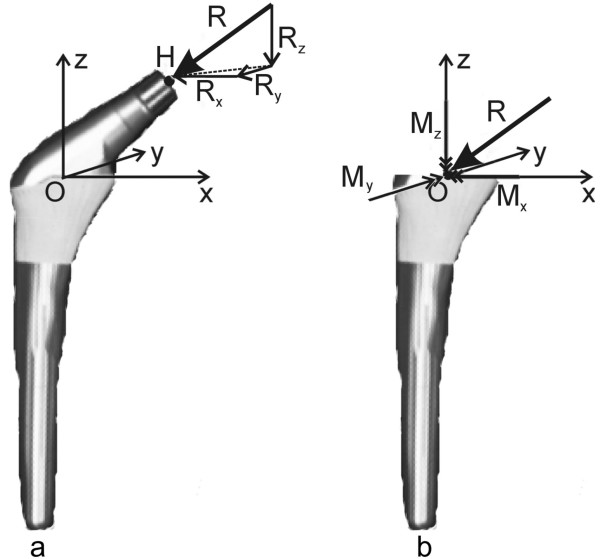
**Loading on a hip prosthesis**. (a) single force $\vec{R}$ on the head (b) equivalent combination of force $\vec{R}$ and moment $\vec{M}$ on the stem. If $\vec{R}$ is considered as a vector with three components (R_x_, R_y_, R_z_), $\vec{M}$ would cause torque around the three axes and is represented by double-arrow-headed vector components (M_x_, M_y_, M_z_). In this study, only M_z _was considered.

Figure 2a shows a prosthesis with an external load $\vec{R}$ on the head H. This situation is of course mechanically equivalent to the situation shown in figure 2b, where the moment $\vec{M}$ in point O is due to the pure force $\vec{R}$ on the head H of the prosthesis (in other words: $\vec{M}$ can be deduced from $\vec{R}$ as: $\vec{M}=\vec{R}\times \vec{HO}$). We will continue with the representation in 2b.

The principle of virtual work says that:

(4) $$\vec{R}\cdot \vec{\delta r_{O}}+\vec{M}\cdot \vec{\delta \theta }+\sum_{i}\vec{F_{i}}\cdot \vec{\delta r_{Ci}}=0$$

With external forces $\vec{R}$, moments $\vec{M}$ and rotations $\vec{\delta \theta }$. Virtual displacement $\vec{\delta r_{Ci}}$ of C_i _can be written as:

(5) $$\vec{\delta r_{Ci}}=\vec{\delta r_{O}}+\vec{\delta \theta }\times \vec{OC_{i}}=\vec{\delta r_{O}}+\vec{\delta \theta }\times \vec{r_{Ci}}$$

Likewise, the real displacement $\vec{\Delta r_{Ci}}$ of C_i _can be written as:

(6) $$\vec{\Delta r_{Ci}}=\vec{\Delta r_{O}}+\vec{\Delta \theta }\times \vec{OC_{i}}=\vec{\Delta r_{O}}+\vec{\Delta \theta }\times \vec{r_{Ci}}$$

The prosthesis stem has six degrees of freedom. Thus, (4) gives rise to six scalar equations, but in this study only the case of pure axial rotation is illustrated. Axial rotation of the prosthesis stem corresponds with a virtual displacement:

(7) $$\vec{\delta r_{O}}=\left(\begin{array}{ccc} 0 & 0 & 0 \end{array}\right)\text{ and }\vec{\delta \theta }=\left(\begin{array}{ccc} 0 & 0 & \delta \theta \end{array}\right)$$

Using (7) in (5) gives:

(8) $$\vec{\delta r_{Ci}}=\left(\begin{array}{ccc} -y_{Ci}\cdot \delta \theta & x_{Ci}\cdot \delta \theta & 0 \end{array}\right)$$

And the set of equations (4) leads to only one non-zero equation:

(9) $$M_{z}-\sum_{i}\left[\left(\frac{EA_{i}}{\ell_{0i}}\cdot \vec{\Delta r_{Ci}}\cdot \vec{n_{i}}\right)\cdot \left(-y_{Ci}n_{ix}+x_{Ci}n_{iy}\right)\right]=0$$

If we assume that the real displacement of the prosthesis due to this load is a rotation around the vertical axis Δ*θ*, we get:

(10) $$\vec{\Delta r_{Ci}}=\left(\begin{array}{ccc} -y_{Ci}\cdot \Delta \theta & x_{Ci}\cdot \Delta \theta & 0 \end{array}\right)$$

and:

(11) $$\Delta \theta =\frac{M_{z}}{\frac{E}{\ell_{0}}\cdot \sum_{i}A_{i}\cdot {\left(-y_{Ci}n_{ix}+x_{Ci}n_{iy}\right)}^{2}}$$

If we define the resistance against axial rotation as the external moment which is needed to make the prosthesis rotate over one radian, this is equal to:

(12) $$\frac{E}{\ell_{0}}\cdot \sum_{i}A_{i}\cdot {\left(-y_{Ci}n_{ix}+x_{Ci}n_{iy}\right)}^{2}$$

Similar expressions can be derived for resistance against subsidence and resistance against inclination of the prosthesis. In this study, only axial rotation is considered because the largest micromotions occur under torsional loading of the prosthesis [13,18]. The resistance against axial rotation is further referred to as antirotation.

An algorithm for reading an STL representation of the prosthesis stem geometry and calculating the resistance against rotation and the estimated rotation Δ*θ *under a torsional moment M_z _was implemented in MATLAB (The Mathworks, Natick, MA, USA). The numerical accuracy of the MATLAB implementation was verified on a simplified model of a rectangular beam with a coarse STL mesh.

### Finite Element model

**Figure 3:**
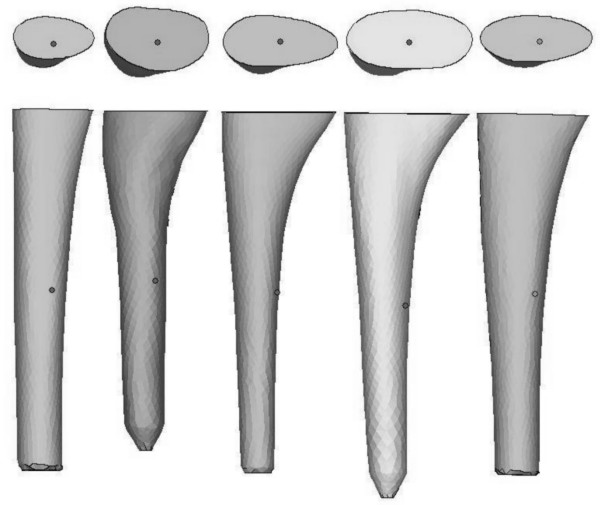
**Upper and anteroposterior view of the five stem geometries**. From left to right: RotaMIN, RotaLOW, RotaMED, RotaHIGH and RotaMAX.

For five stem designs, a FE model of the bone-implant complex was built, that aimed at replicating the simplified assumptions of the analytical model as good as possible. The stems were chosen in such a way that they span a wide range of antirotation values, based on the analytical model. STL-files of the stem geometries were provided by the university hospital orthopaedics department, which allowed calculation of the resistance against axial rotation with the proposed analytical model. Export of stem geometries in STL format was available as a utility within the system software of the IMP system used in the Leuven university hospital. The antirotation values for all five stems are shown in table 1. The names of the stems refer to their mutual ranking, based on the antirotation values; the resistance against rotation increases from left (RotaMIN) to right (RotaMAX) in table 1. The stem geometries are shown in figure 3.

**Table 1:** Resistance against axial rotation for the five stem designs, calculated with the analytical model.

	**RotaMIN**	**RotaLOW**	**RotaMED**	**RotaHIGH**	**RotaMAX**
**Resistance against axial rotation [Nm/rad]**	3.40E+05	4.66E+05	7.81E+05	9.87E+05	1.25E+06

In order to relate the results from the FE simulations with the analytical model, the conditions of the analytical model explained above have to be fulfilled in the FE simulations as well. By applying a uniform extrusion around the prosthesis, a bone layer of homogeneous thickness of 10 mm was created. To comply with the condition of complete contact between bone and prosthesis, the cavity in the bone was obtained directly from the prosthesis volume. Frictionless touching contact was defined between the prosthesis and the bone. Finally, the constraints in the FE model prohibit all movements of the outer surface of the bone. Linear tetrahedral 4-node elements (TET4 type) were used to build the FE models and coincident nodes were used at the bone-stem interface. The resulting models had a number of elements ranging from 46000 to 53000. The element size was 3 mm for the outer surface of the bone mantle, and 2 mm for the prosthesis surface and the inner bone mantle (i.e. in contact with the stem). Internal coarsening was used. Mesh refinements were applied at edges and where stress concentrations were expected.

Calculations were performed with MARC/Mentat FE software (MSC.Software, NL). Bone and prosthesis were assumed to have a Poisson's ratio of 0.3; the Young's modulus used for the titanium stems was 114000 MPa; the bone was assumed to be trabecular bone and was given a Young's modulus of 233 MPa [29]. The low stiffness of trabecular bone results in larger stem displacements, which improves the relative accuracy of the FE results.

Both titanium and bone material were assumed isotropic and linear elastic. For comparison with the analytical model, all five prostheses were subjected to internal-rotation torsional moments of 4 Nm, 10 Nm and 20 Nm along the z-axis. The maximum load of 20 Nm corresponds to the highest torsional loads to which a hip prosthesis is exposed, i.e. under stair-climbing [18]. The corresponding rotation angle was obtained for each load case. Based on the antirotation values of the stems, the rotation angles predicted by the analytical model could also be calculated under the same torsional loads. The run time of an FE analysis ranged from 45 minutes up to 60 minutes. The run times of the MATLAB implementation of the analytical model were less than 10 seconds.

## Results

**Figure 4:**
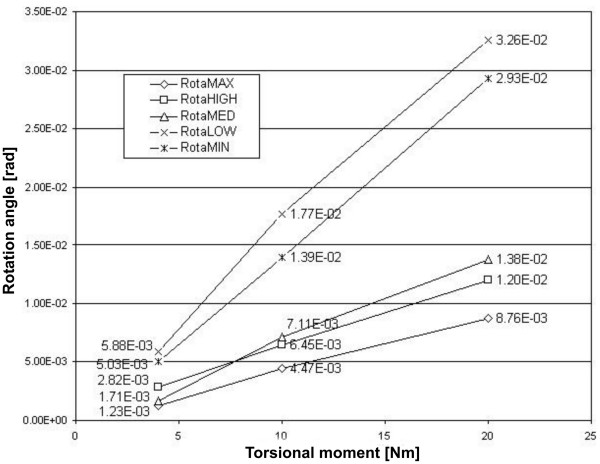
**Rotation angle as a function of the applied torsional load**. Calculated for the finite element models of the five stem geometries implanted in a trabecular bone mantle.

The progress of the stem rotation about the z-axis as a function of the applied torsional load, obtained from the FE simulations, is shown in figure 4 for all five prostheses. The lines connecting the data are merely for visualisation and have no further meaning.

The stability order of the stems resulting from the FE simulations is in fairly good accordance with their ranking based on the antirotation values; RotaMAX exhibits the smallest rotation angles, RotaHIGH is the second most stable stem, closely followed by RotaMED. Only RotaLOW and RotaMIN have switched places in the stability ranking obtained with FEM: for RotaMIN, smaller rotation angles are found than for RotaLOW, which is the least stable stem. The stability ranking of the stems is independent of the applied torsional load, except that RotaMED has a smaller rotation angle at 4 Nm than RotaHIGH. An approximately linear progress of the rotation angle with the applied torsional moment was found for all five stems over the observed loading interval.

The expected inducible displacements were also calculated with the analytical model, under the same torsional loads; the rotation angles could be derived from the antirotation values, assuming a linear relationship between the applied load and the resulting rotation of the stem. For ease of comparison, the rotation angles calculated with the analytical model as well as those obtained from the FE simulations are shown in table 2. When comparing the results for both assessment techniques, the rotation angles predicted by the analytical model turn out to be two to three orders of magnitude smaller than those obtained using FEM.

**Table 2:** Rotation angles*, obtained with FEM and with the analytical model.

	**Moment [Nm]**	**RotaMIN**	**RotaLOW**	**RotaMED**	**RotaHIGH**	**RotaMAX**
**FEM**	4	5,03E-03	5,88E-03	1,71E-03	2,82E-03	1,23E-03
	10	1,39E-02	1,77E-02	7,11E-03	6,45E-03	4,47E-03
	20	2,93E-02	3,26E-02	1,38E-02	1,20E-02	8,76E-03
	
**Analytical model**	4	1,18E-05	8,58E-06	5,12E-06	4,05E-06	3,20E-06
	10	2,94E-05	2,15E-05	1,28E-05	1,01E-05	8,00E-06
	20	5,88E-05	4,29E-05	2,56E-05	2,03E-05	1,60E-05

*Angles expressed in radians, for all five stems, under torsional loads of 4 Nm, 10 Nm and 20 Nm.

## Discussion

In this study, a mathematical formulation is proposed to numerically predict the potential primary stability of cementless hip stems. This analytical approach is based upon the principle of virtual work and a straightforward mechanical model. The only input needed is an STL-file of the stem, which can easily be obtained from most CAD-systems. In this way, the model provides a fast and inexpensive measure for the expected primary stability.

Such quantification of the primary stability might be useful for several purposes. The range of micromotions measured under similar loading conditions varied considerably among different in vitro studies [12,13,16,17]. Considering this large experimental variability, several authors have indicated that a theoretical approach could be useful in determining the potential primary stability of a certain hip stem. Also in preoperative planning systems for THRs, quantification of the primary stability might be an excellent measure for the expected long-term results. Fast assessment of the primary stability might be even more beneficial for IMP-systems, like the one used at our university hospital orthopaedics department [28]. In this case, the prosthesis is designed intraoperatively, based on the geometry of the reamed cavity. This necessitates a fast stability quantification of the proposed stem design, to limit the operation time.

Several other methods have been suggested to quantify the primary stability of a cementless stem without the need for measurements [8,9,21]. However, to our knowledge, all the alternatives reported in literature rely on the use of FEM. The possibilities with FEM are very extensive: it allows a complete mapping of the interface micromotion [30,31] and the effect of the surrounding bone quality can be taken into account using a FE model of the proximal femur [4]. But the use of FEM also has some important drawbacks: the introduction of FEM in clinical practice would require high performance computing hardware to keep the runtimes within acceptable limits. In combination with the needed specialised software, this would result in much more expensive THRs. Another important consideration is that clinical personnel usually do not have sufficient expertise in computational mechanics. Therefore, emphasis must be placed upon developing a computer interface that is easy to use for the surgeon [10]. The analytical model presented in this study could be considered as a first attempt to provide a theoretical measure for the primary stability of cementless hip stems without using FEM. This implies that the proposed analytical approach does not suffer from the main disadvantages of FEM: it provides a fast and inexpensive measure of the primary stability, and the required human-computer interaction is very limited. The drawback is that the stability feedback obtained from the analytical model is rather limited and not quantitative: critical information concerning primary stability might be lost due to the strong simplifications of the model, and this could compromise the relevance of the feedback. This latter concern is addressed in this study: for five stem designs, the resistance against axial rotation was assessed using FEM on the one hand and the analytical model on the other.

With respect to the FE models, the analytical model predicts a very stiff stability behaviour: stem rotation was found to be two to three orders of magnitude smaller for all loadcases, compared with the FE simulations. The rotation values obtained with the FE models correspond to displacements that are in the same order of magnitude as those found in literature [9,12,13,16,17]. For instance, under a simulated torsional load of 20 Nm, the largest displacements at the stem/femur interface varied between 216 *μ*m (for RotaMAX) and 684 *μ*m (for RotaLOW) for all five stems. This means that the stability behaviour predicted by the analytical model is unrealistically stiff. Although the FE models used in this study were built according to the simplified assumptions of the analytical model, some important differences remain that can explain a stiffer behaviour of the analytical model; first of all, shearing deformation of the bone is not considered in the analytical model: it assumes that the bone surrounding the stem will only deform under radial compression, and that the resulting resistive force is proportional to the compression of the bone. It is however to be expected that some shear deformation of the bone will also occur, partially because the inner surface of the bone mantle will rotate with respect to the outer surface, due to the transfer of the torsional load. Exclusion of the shearing of the bone might result in a much stiffer behaviour of the bone-implant complex.

A second important difference concerns the displacement of the stem: the analytical model assumes that the stem displacement under torsional load will be a pure rotation about a vertical axis through the centre of gravity of the stem. However, the FE simulations have shown that the stem displacement is more complex: for all cases, the vertical rotation axis is shifted along the mediolateral axis with respect to the centre of gravity, and in some cases some tilting of the stem was also found. Preliminary FE tests have shown that allowing the stem only to rotate about a vertical axis through the centre of gravity results in much smaller simulated stem rotations. The effect of both excluding the shearing deformation of the bone and restricting the displacement of the stem to a rotation about the centre of gravity will be addressed in the continuation of the research.

The stability order of the stems was in reasonable agreement for both methods; the analytical model predicts the same stability ranking as the FE simulations, except that RotaMIN and RotaLOW switched places. However, the difference in the antirotation values for both stems is small. Figure 4 also shows a gap in the results between the two least stable stems, RotaMIN and RotaLOW, and the other three stems. This gap is also present in the antirotation values of the stems and in the resulting rotation angles. Based on these results, the analytical model seems to be useful as a relative measure for the primary stability of cementless hip stems. In a recent study by Prendergast et al., inducible displacements were measured for four stem designs in in vitro experiments and a stability ranking of the stems was based upon these measurements [20]. It was found that this stability ranking of the stems correlated well with their clinical performance. Similarly, it might be useful to seek a relation between the stability ranking of stems based on the analytical model and their clinical performance; this might result in a threshold antirotation value that can be used to distinguish between stems with sufficient and insufficient primary stability; new stem designs with an antirotation value lower than this threshold could then be dismissed without the need for measurements. However, for the time being, these are only speculations; in order to validate the analytical model, more stems need to be included in the study, and the antirotation values should be compared with in vitro measurements of the primary stability of implanted stems.

Indeed, the stability order of the stems might be different when more realistic models of the bone-implant complex or truly implanted stems are observed: instead of a perfect fit-and-fill of the cavity, gaps will occur at the bone-implant interface; the contact between the prosthesis and the bone is not frictionless, but frictional forces will considerably contribute to the resistance against axial rotation [32]; the prosthesis makes contact with cortical as well as trabecular bone, and the bone mantle does not have a homogeneous thickness and stiffness; the outer surface of the bone mantle is not rigidly fixed, it can also deform. All of these simplified assumptions, which are considered in this study, might result in an incorrect stability ranking of the stems. In that case, the analytical model should be refined, as to eliminate the simplification(s) that cause the ranking error. In a combined experimental and FE modelling study on the rotational stability of cementless THR stems [33], FE-predicted rotational micromotions were 2–20 times larger with a friction coefficient f = 0.3 than with f = 0 and this suggests that consideration of friction should be a priority when the analytical model is to be refined.

## Conclusion

In conclusion, it was found that the analytical model predicts an unrealistically stiff stability behaviour. Although the FE models used in this study aimed at replicating the simplified assumptions of the analytical model, some important differences occurred with respect to the stability behaviour: stem displacement resulting from a pure torsional load is not always a pure rotation about a vertical axis, as is assumed by the analytical model. Instead, the displacement path followed by the stem is imposed by the shape of the contact surface. Secondly, bone deformation is modelled as pure radial compression in the analytical model. This assumption will also significantly reduce the predicted displacements, since shear deformation of the bone is excluded. Both of these issues will be addressed in the continuation of the research.

Nevertheless, the analytical model seems to be useful as a comparative tool for the primary stability of cementless hip stems. The stability ranking obtained for real implanted stems might differ from the ranking obtained with the analytical model, due to the strong simplifications on which the model is based. Future research should therefore consider more realistic models of the bone-implant complex or in vitro measurements of the primary stability. If necessary, further refinements should be made to the model to eliminate the simplifications that cause errors in the stability ranking.

## Competing interests

The authors declare that they have no competing interests.

## Authors' contributions

MEZ designed and analyzed the first versions of the finite element models and drafted the initial manuscript. LL developed the analytical model for hip stem stability, with input from GVDP and SVNJ. NS refined and re-analyzed the finite element models and extended the manuscript accordingly. MM provided clinical background and contributed to the interpretation from a practitioner's view. GVDP and SVNJ conceived and coordinated the study as a comparison of analytical and finite element modeling to predict stem stability. Interpretation of the comparison between analytical and FE model results was a joint effort by MEZ, NS, LL, GVDP and SVNJ. All authors read and approved the final manuscript.
